# Transcriptional regulation of flavonoid biosynthesis in *Artemisia annua* by AaYABBY5

**DOI:** 10.1038/s41438-021-00693-x

**Published:** 2021-12-01

**Authors:** Sadaf-Ilyas Kayani, Qian Shen, Saeed-ur Rahman, Xueqing Fu, Yongpeng Li, Chen Wang, Danial Hassani, Kexuan Tang

**Affiliations:** Joint International Research Laboratory of Metabolic and Developmental Sciences, Plant Biotechnology Research Center, Fudan-SJTU-Nottingham Plant Biotechnology R&D Center, School of Agriculture and Biology, Shanghai Jiao Tong University, 200240 Shanghai, China

**Keywords:** Plant molecular biology, Metabolic engineering, Secondary metabolism

## Abstract

*Artemisia annua* is a medicinal plant rich in terpenes and flavonoids with useful biological activities such as antioxidant, anticancer, and antimalarial activities. The transcriptional regulation of flavonoid biosynthesis in *A. annua* has not been well-studied. In this study, we identified a YABBY family transcription factor, AaYABBY5, as a positive regulator of anthocyanin and total flavonoid contents in *A. annua*. AaYABBY5 was selected based on its similar expression pattern to the phenylalanine ammonia lyase (*PAL*), chalcone synthase (*CHS*), chalcone isomerase (*CHI*), and flavonol synthase (*FLS*) genes. A transient dual-luciferase assay in *Nicotiana bethamiana* with the AaYABBY5 effector showed a significant increase in the activity of the downstream *LUC* gene, with reporters *AaPAL*, *AaCHS, AaCHI*, and *AaUFGT*. The yeast one-hybrid system further confirmed the direct activation of these promoters by AaYABBY5. Gene expression analysis of stably transformed *AaYABBY5* overexpression, *AaYABBY5* antisense, and control plants revealed a significant increase in the expression of *AaPAL*, *AaCHS, AaCHI, AaFLS, AaFSII, AaLDOX*, and *AaUFGT* in *AaYABBY5* overexpression plants. Moreover, their total flavonoid content and anthocyanin content were also found to increase. *AaYABBY5* antisense plants showed a significant decrease in the expression of flavonoid biosynthetic genes, as well as a decrease in anthocyanin and total flavonoid contents. In addition, phenotypic analysis revealed deep purple-pigmented stems, an increase in the leaf lamina size, and higher trichome densities in *AaYABBY5* overexpression plants. Together, these data proved that AaYABBY5 is a positive regulator of flavonoid biosynthesis in *A. annua*. Our study provides candidate transcription factors for the improvement of flavonoid concentrations in *A. annua* and can be further extended to elucidate its mechanism of regulating trichome development.

## Introduction

*Artemisia annua* is a renowned plant rich in terpenes and flavonoids. Flavonoids are polyphenolic plant secondary metabolites. Anthocyanins, flavonols, flavanols, and proanthocyanidins (PAs) or condensed tannins are the major classes of flavonoids. These compounds show differential expression patterns according to plant growth or developmental stages and in a species-specific manner^1^. Flavonoids perform various functions in plants, including antioxidant activity, protection against UV light, defensive responses against plant pathogens, activation of nodulation genes in legumes, fertility, and auxin transport^2^. Flavonoids are also beneficial for human health in many aspects as a nutritional source, as well as for the treatment of various diseases.

Increasing evidence recommends using the dried leaves of *A. annua*, which contain both artemisinin and flavonoids, to cure malaria more effectively than using only artemisinin^3^. A recent study showed that the combination of kaemferol and artesunate exerts potent antimalarial activity synergistically in mice infected with malarial plasmodium^4^. In a recent study, the mechanism for increased bioavailability of artemisinin when using dried leaves that supply artemisinin and flavonoids synergistically was shown^5^. A comparative study indicated the parallel biosynthesis of flavonoids and artemisinin at three growth stages, except for a few flavonoids that showed altered behavior. Furthermore, flavonoids of *A. annua* also exhibit efficacious antioxidant effects^6^, strong anti-inflammatory properties^7^, and potent anticancer characteristics^8^. To cure cancer, various flavonoids of *A. annua* have been reported to synergize with many anticancer drugs. The reported flavonoids that aid anticancer drugs include apigenin, eupatin, luteolin, silyben, kaempferol, and quercetin^9–14^. In a recent study, potent anti-inflammatory activities of the flavonoids of *A. annua* were reported^7^.

**Fig. 1:**
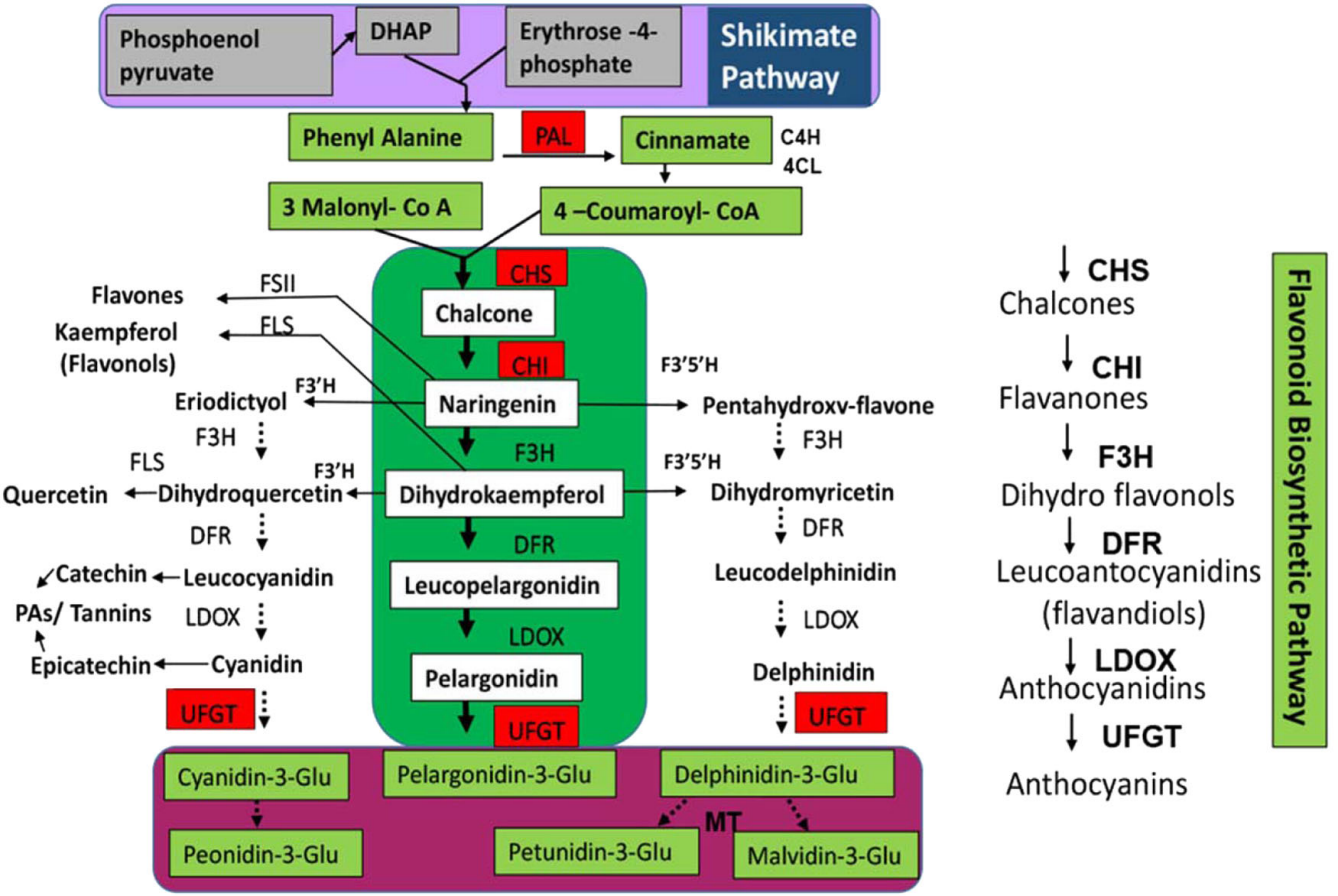
The flavonoid biosynthetic pathway in *Artemisia annua*. PAL1 phenylalanine ammonia lyase, C4H cinnamate 4-hydroxylase, 4CL 4-coumarate-CoA ligase, CHS chalcone synthase, CHI chalcone isomerase, F3H flavanone-3-hydroxylase, F3’H flavonoid 3’-hydroxylase, F3'5’H flavonoid 3’,5’-hydroxylase, FSII flavone synthase II, FLS flavonol synthase, DFR dihydroflavonol reductase, LDOX leucoanthocyanidin dioxygenase, UFGT UDP-glucose flavonoid 3-O-glucosyl transferase, MTs methyltransferases are key enzymes involved in the pathway

*A. annua* can produce ~40 different flavonoids, which may vary depending upon the cultivar or Line^15^. Despite the massive abundance of flavonoid varieties in *A. annua* and their significant therapeutic potential, the flavonoid biosynthetic pathway and its transcriptional regulation have not been well-elucidated. A few studies have been reported on the regulation of flavonoid biosynthesis in *A. annua*^16,17^. In other plant species, including *A. thaliana*, the flavonoid biosynthetic pathway has been thoroughly studied^18^ (Fig. 1). The upstream shikimate pathway provides the precursor amino acid phenylalanine, which is catalyzed by phenylalanine ammonia lyase (PAL), to generate cinnamate^19^. Cinnamate is then transformed into 4-coumaroyl-CoA in the presence of cinnamate 4-hydroxylase (C4H) and 4-coumarate-CoA ligase (4CL)^20^. The first committed step toward flavonoid biosynthesis involves 3x malonyl-CoA and 1×4-coumaroyl-CoA to generate chalcones (the basic skeleton of all flavonoids) in a condensation reaction catalyzed by chalcone synthase (CHS)^21,22^. In the next step, chalcone is isomerized to the flavanone naringenin in the presence of chalcone isomerase (CHI)^23^. In the following step, which generates dihydroflavonones, naringenin is oxidized by flavanone-3-hydroxylase (F3H) to yield dihydrokaempferol, which is subsequently hydroxylated by flavonoid 3’-hydroxylase (F3’H) or flavonoid 3’,5’-hydroxylase (F3'5’H), producing dihydroquercetin or dihydromyricetin, respectively^24^.

F3’H or F3'5’H may also directly perform the hydroxylation of naringenin to yield flavonones called eriodictyol and pentahydroxy-flavanone, which upon hydroxylation generate dihydroquercetin and dihydromyricetin, respectively. Next, three dihydroflavonols are converted to anthocyanidians in a two-step reaction catalyzed by dihydroflavonol reductase (DFR) and leucoanthocyanidin dioxygenase (LDOX), successively. In the first step, DFR is involved, which converts dihydroquercetin to leucocyanidin, dihydrokaempferol to leucopelargonidin, and dihydromyricetin to leucodelphinidin. The second step is involved in the LDOX-mediated catalytic oxidation of leucocyanidin to cyanidin, leucopelargonidin to pelargonidin, and leucodelphinidin to delphinidin. In the final step, cyanidin, pelargonidin, and delphinidin are glycosylated by the enzyme UDP-glucose flavonoid 3-*O*-glucosyl transferase (UFGT) to produce colorful and stable compounds called anthocyanins^25^. Anthocyanin:cyanidin-3-glucoside may be further converted to peonidin-3-glucoside, and delphinidin-3-glucoside is converted to petunidin or malvidin-3-glucoside after being methylated by methyltransferases (MTs). Molecules called tannins or PAs are generated by the enzymes leucoanthocyanidin reductase (LAR) and anthocyanidin reductase (ANR), which catalyze the reduction of leucocyanidin to catechin or cyanidin to epicatechin, respectively.

Recently, several genes from the *A. annua* flavonoid biosynthetic pathway, including chalcone isomerase (*AaCHI*) phenylalanine ammonia lyase (*AaPAL1*), flavanone-3-hydroxylase (*AaF3*′*H*), and *AaFLS1*, have been cloned and characterized^16,17,26,27^. A recent study showed that the overexpression of *AaCHI* in *A. annua* provides not only increased levels of flavonoids but also artemisinin^27^. However, the transcriptional regulation of flavonoid biosynthesis in *A. annua* has not been well-elucidated. The overexpression of *MYC2* in *A. annua* resulted in an increased level of both anthocyanins and artemisinin^28^. Most of the R2R3-MYB genes have been shown to play regulatory roles in flavonoid biosynthesis in *A. annua*. Recently, the MYB family transcription factor AaTAR2 from *A. annua* was reported to synergistically regulate both artemisinin and flavonoids^29^; however, the mechanism of the regulation of flavonoid biosynthesis was not found. In plants, flavonoid biosynthesis is tightly regulated by MBW complexes that interact with late biosynthetic genes. In *Arabidopsis thaliana*, this ternary complex comprises specific R2R3-MYB (PAP1, PAP2, MYB113, or MYB114) and bHLH transcription factors (GLABRA 3, GL3 or Enhancer of GLABRA 3, ECL3) that interact with WDR proteins (TTG1; TRANSPARENT TESTA GLABRA 1)^30–34^. However, these complexes have not yet been identified in *A. annua*. The identification of transcription factors that regulate flavonoid biosynthesis (by direct means or via indirect interactions), the discovery of MBW complexes and their transcriptional control are important to obtain a better understanding of flavonoid biosynthesis in *A. annua*.

The YABBY gene family is a small family of transcription factors that are specifically found in only seed plants. Due to their small group and their role in primary and secondary plant metabolism, they are of great interest to researchers. Over the last decade, several studies have been conducted on YABBY proteins; however, the function of YABBY family transcription factors in flavonoid biosynthesis in *A. annua* was not previously characterized. The characteristic features of the YABBY family are the N-terminal C_2_C_2_ zinc finger-like domain and C-terminal HMG domain^35,36^. The zinc finger domain mediates protein–protein interactions, whereas the YAB domain mediates DNA binding. YABBY protein complexes generate homo or heterodimers between the YABBY proteins and form complexes with other proteins^37^. Angiosperms such as Arabidopsis contain six YABBY genes in their genomes^35,36^, which are classified into six groups, FIL + YAB3, YAB2, YAB5, CRC, and INO, and are found to be transcriptional regulators^38,39^.

The primary function of YABBYs in plants is the regulation of lamina outgrowth and leaf development^40^. YABBY proteins interact with LEUNIG or its homologs, and YAB-LUC complexes regulate adaxial cell polarity in leaves and the initiation of the apical meristem in shoots^41^. It was first thought that these transcription factors were limited to the regulation of primary metabolic processes in plants, and massive data on YABBY-mediated leaf development in various seed plants are available. However, during the last decade, a few studies have reported the role of YABBY transcription factors in secondary metabolism, such as the regulation of anthocyanins and glucosinolate through *AtFIL* in *A. thaliana* and the control of monoterpene biosynthesis by *MsYABBY5* in *Mentha spicata*^42–44^. YABBY transcription factors also work in response to various stresses and plant defense responses; for example, Arabidopsis *YABBY10* is involved in drought and salt stress responses^45^, and *AtFIL* knockout lines provide good resistance to bacterial infections^42^.

In our previous study, we cloned and characterized a YABBY gene; AaYABBY5 is a positive regulator of artemisinin biosynthesis that directly binds to the promoters of *AaCYP71AV1*, and *AaDBR2,* activates their gene expression and results in a significant increase in the concentration of artemisinin. In this study, we elaborated on the role of AaYABBY5 in the biosynthesis of flavonoids, including anthocyanins, and their regulation. AaYABBY5 was selected for this study based on its similar expression pattern to that of the *PAL, CHI*, and *FLS* genes. YABBY5 was found to be a direct activator of promoters of the *PAL, CHI, CHS*, and *UFGT* genes in the transient *Nicotiana benthamiana* infiltration system and yeast transformation system, respectively. In addition, comparative analysis of transgenic *A. annua* plants that overexpressed the *AaYABBY5* ORF, overexpressed the antisense RNA of *AaYABBY5*, plants transformed with empty pHB vector and untransformed/wild-type plants revealed a significant increase in the transcript levels of *AaPAL*, *AaCHI*, *AaCHS*, *AaFLS, AaFSII, AaLDOX*, and *AaUFGT*, as well as enhanced flavonoid and anthocyanin production in *AaYABBY5* overexpression plants but a significant decrease in gene expression, flavonoids, and anthocyanins in *AaYABBY5* antisense plants compared to the control plants.

To our knowledge, in *A. annua*, the mechanism of the transcription factor-mediated direct activation of flavonoid biosynthesis genes has not been studied before. Previous data do not explain how flavonoids are regulated at the molecular level. Our study provided evidence of direct regulation of the *PAL* gene, which is present upstream of the flavonoid biosynthetic pathway; *AaCHS* and *AaCHI*, which regulate the first committed step of flavonoid biosynthesis and the next preceding step, respectively; and *UFGT*, which is involved in the conversion of precursor anthocyanidin molecules to anthocyanins. The present study broadens the knowledge on the direct regulation of flavonoids by AaYABBY5 in *A. annua*. To our knowledge, we provide the first molecular mechanism of flavonoid regulation in *A. annua*.

## Results

### Flavonoid biosynthetic genes contain YABBY-binding motifs

**Fig. 2:**
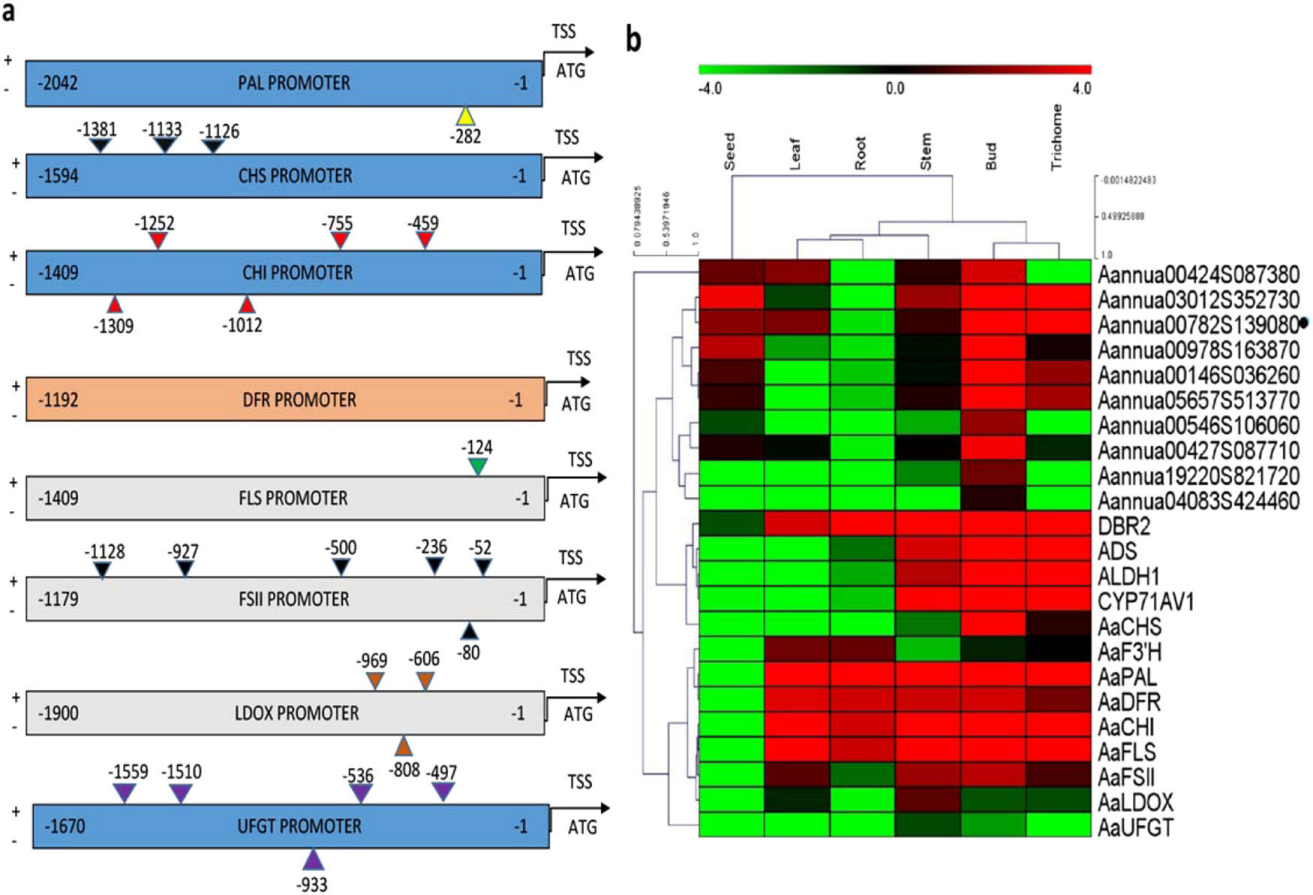
Bioinformatic analysis of the promoters of flavonoid biosynthetic genes, as well as the expression profile of YABBY family genes and flavonoid-regulating genes in *A. annua*. **a** Putative YABBY-binding sites predicted by PLANTPAN3.0 are shown. Positions on plus and minus strands are represented by numbers above and below the promoter sequence, respectively. YABBY-binding sites were found in all promoters except the *DFR* promoter sequence, for which no predicted YABBY-binding site was predicted. **b** Heatmap showing the expression profile of YABBY family genes, as well as flavonoid biosynthetic genes in six tissues. The color scale at the top represents the RPKM (reads per kilobase per million mapped reads) values. AaYABBY5 (marked with a black dot) was selected as a potential transcription factor that might regulate flavonoid biosynthesis because of its similar expression pattern to the important flavonoid biosynthetic genes

The cloned promoter sequences of flavonoid biosynthetic genes used in the present study, *AaPAL*, *AaCHS, AaCHI*, *AaFLS*, *AaFSII*, and genes regulating anthocyanins *AaDFR, AaLDOX*, and *AaUFGT*, were analyzed for putative YABBY-binding sequences^37^ using PlantPAN 3.0 (PlantPAN; http://PlantPAN.itps.ncku.edu.tw). Recent work, including ChIP and RNA-seq studies on YABBY-binding motifs present in soya bean and protein-binding microarrays in *A. thaliana*, has shown that these sites vary greatly among different species. YABBY-binding motifs are represented by AT-rich sites in Arabidopsis with consensus binding sequences defined as AATNATAA and AATNATTA. The homologous YABBY-binding motifs found in the promoter sequences are shown in Fig. 2a, with the positions marked by numbers. Except for *DFR*, YABBY-binding motifs were identified in all promoters.

### Global expression profile of YABBY family genes and selection of AaYABBY5 as a potential transcription factor regulating flavonoid biosynthesis

The transcriptome data of six different tissues of *A. annua* were previously generated by our lab^46^. Plant secondary metabolites are usually synthesized in a species- or tissue-specific manner, and secondary metabolites, including flavonoids, are synthesized in trichomes^47^. To identify potential YABBY family genes that might regulate flavonoid biosynthesis, a heatmap was constructed to compare the expression of YABBY genes and flavonoid biosynthetic genes across tissues; trichomes, buds, stems, roots, leaves, and seeds (Fig. 2b).

We found two YABBY genes clustered with flavonoid pathway genes, showing higher expression in trichomes and buds. Among the YABBY family genes, AaYABBY5 (marked with a black dot) was found to be a candidate transcription factor that showed a transcription profile parallel to that of *PAL, CHI, DFR*, and *FLS* and showed higher expression in trichome, bud, stem, and leaf tissues. In our previous findings, we found that AaYABBY5 regulates *DBR2* and *CYP71AV1* that are involved in the artemisinin biosynthetic pathway^48^. Here, we found that *AaYABBY5*, *DBR2*, and *CYP71AV1* showed similar expression patterns, i.e., higher expression in trichomes and buds, with a progressive decline in their expression in the stem tissues.

As this study focused on regulating flavonoid regulation, we hypothesized that AaYABBY5 might regulate *PAL*, *CHS*, *CHI*, and *FLS*, which showed similar expression patterns in trichomes and/or buds. Interestingly, *PAL* and *DBR2* (the YABBY5 target gene) showed similar expression in trichomes, buds, stems, roots, and leaves. *DFR* and *FSII* expression was found more in buds than in trichomes. *F3’H* expression was very different from the other genes present in the flavonoid pathway. LDOX and UFGT are enzymes that are involved in anthocyanin biosynthesis. In *A. annua*, anthocyanins are limited to stem tissues^28^, and the expression of *LDOX* and *UFGT* was found to be higher in stem tissues than in other tissues. In a previous study, real-time PCR analysis of different tissues revealed that *AaYABBY5* transcripts are also found in the stem tissues of *A. annua*,^48^; therefore, we speculated that it might also regulate the *LDOX* and/or *UFGT* genes. Overall, it was speculated that YABBY5 might regulate *PAL*, *CHI*, *CHS*, and *FLS*, which are involved in early flavonoid biosynthesis, and *LDOX and UFGT*, which are present in the late flavonoid (anthocyanin) pathway. Further experiments were carried out to test this hypothesis.

### AaYABBY5 significantly activates the promoters of *AaPAL*, *AaCHI*, *AaCHS*, and *AaUFGT* in transiently transformed *N. benthamiana*

**Fig. 3:**
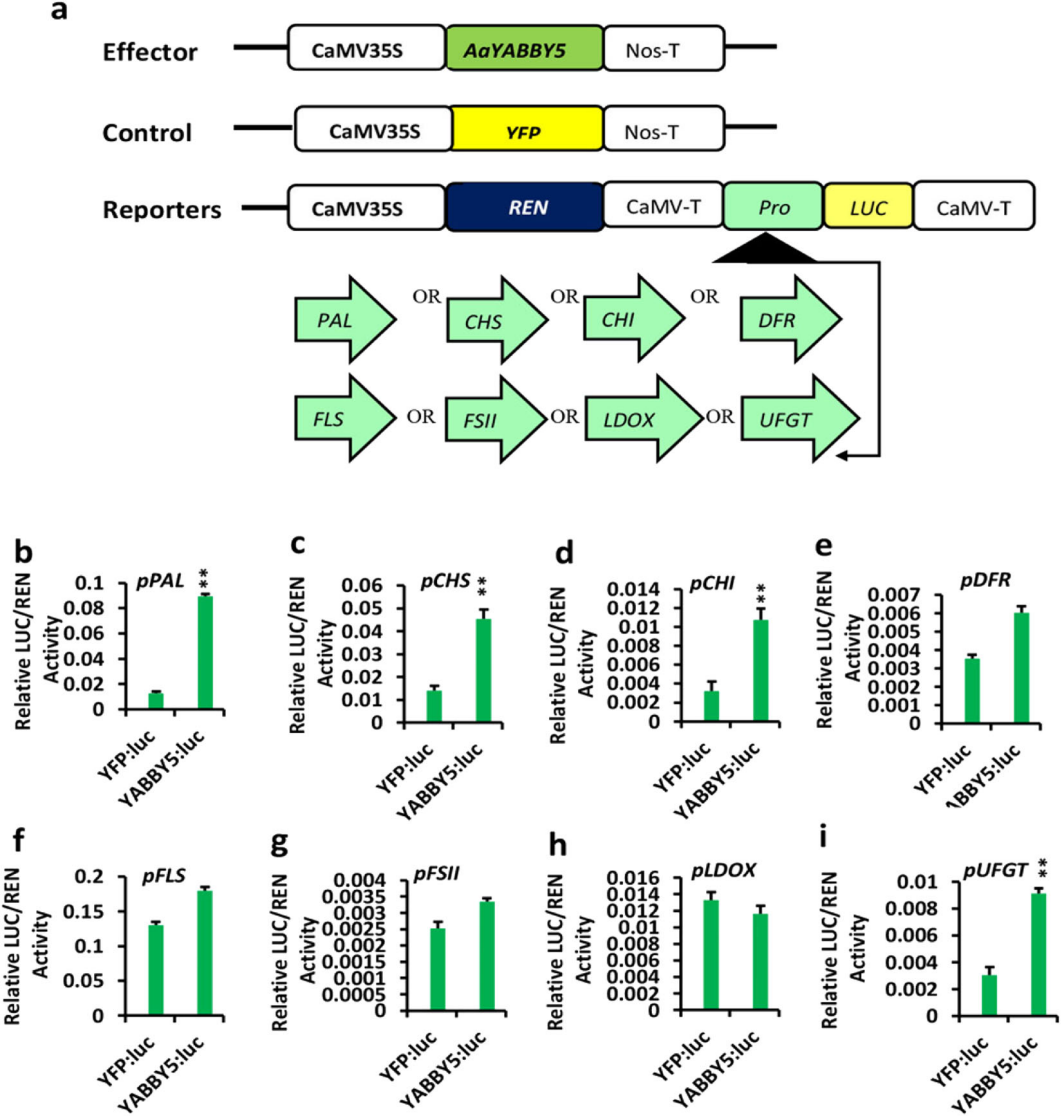
Transient dual-luciferase reporter assay. **a** Schematic representation of constructs used to prepare effector and reporter strains. The *AaYABBY5* open-reading frame fused to the yellow fluorescent protein (YFP-N) in pEG104 was used as the effector. YFP-N was used as a negative control. Promoters of *PAL, CHS, CHI, DFR, FLS, FSII, LDOX*, and *UFGT* were fused with the *LUC* gene at its N-terminus as reporter constructs. From **b**–**i** showing relative LUC activities obtained for each combination of the reporter with effector. Significant increases were found with the *PAL*, *CHI*, *CHS*, and *UFGT* promoters. Data show the mean values ± SD of four independent infiltrations. Error bars show the standard deviation for *n* = 4. **P < 0.01. *P < 0.05. The gene sequence of AaYABBY5 can be found in NCBI GenBank under accession number MK675289. The promoter sequences of *DFR, PAL, CHS, CHI, FLS, FSII, LDOX, and UFGT* have been submitted to the National Center for Biotechnology Information (NCBI), and accession numbers have been assigned as MW558943, MW558944, MW915581, MW558945, MW464242, MW464241, MW464239, and MW464240, respectively

Knowing putative YABBY-binding sites in the *AaPAL*, *AaCHI*, *AaCHS*, *AaDFR, AaFLS, AaFSII, AaLDOX*, and *AaUFGT* promoter sequences, a dual-luciferase assay was performed, where *AaYABBY5* inserted in pEarleyGate 104-YFP was used as an effector and promoter sequences inserted into pGreenII 0800-LUC were used as reporters (Fig. 3a). Equal-sized infiltrated leaf discs for each combination of the reporter with the effector AaYABBY5 were analyzed by commercially available dual-LUC reagents (Promega, USA). Values greater than a twofold increase were taken into consideration, and lower values were negated. A significant increase in relative LUC/REN values was found for the *AaPAL*, *AaCHS*, *AaCHI*, and *AaUFGT* promoters. AaYABBY5 exhibited a 7.4-fold increase in the activity of the *PAL* promoter, a 3.2-fold increase in the activity of the *CHS* promoter, a 3.4-fold increase in the activity of the *CHI* promoter and a three fold increase in the activity of the *UFGT* promoter (Fig. 3b–d, i). The fold change was calculated from comparative values of each effector/reporter, and an empty vector was used as a negative control/reporter. The increase in the LUC/REN values corresponds to the intensity of LUC signals driven by the respective promoters in the presence of the AaYABBY5 protein. Based on these results, it was hypothesized that AaYABBY5 could activate these promoter sequences in *A. annua* either directly, by binding to putative YABBY-binding motifs, or indirectly, through some protein–protein interactions.

### AaYABBY5 directly binds to promoter regions of *AaPAL, AaCHS, AaCHI*, and *AaUFGT* in the EGY48 yeast strain

**Fig. 4:**
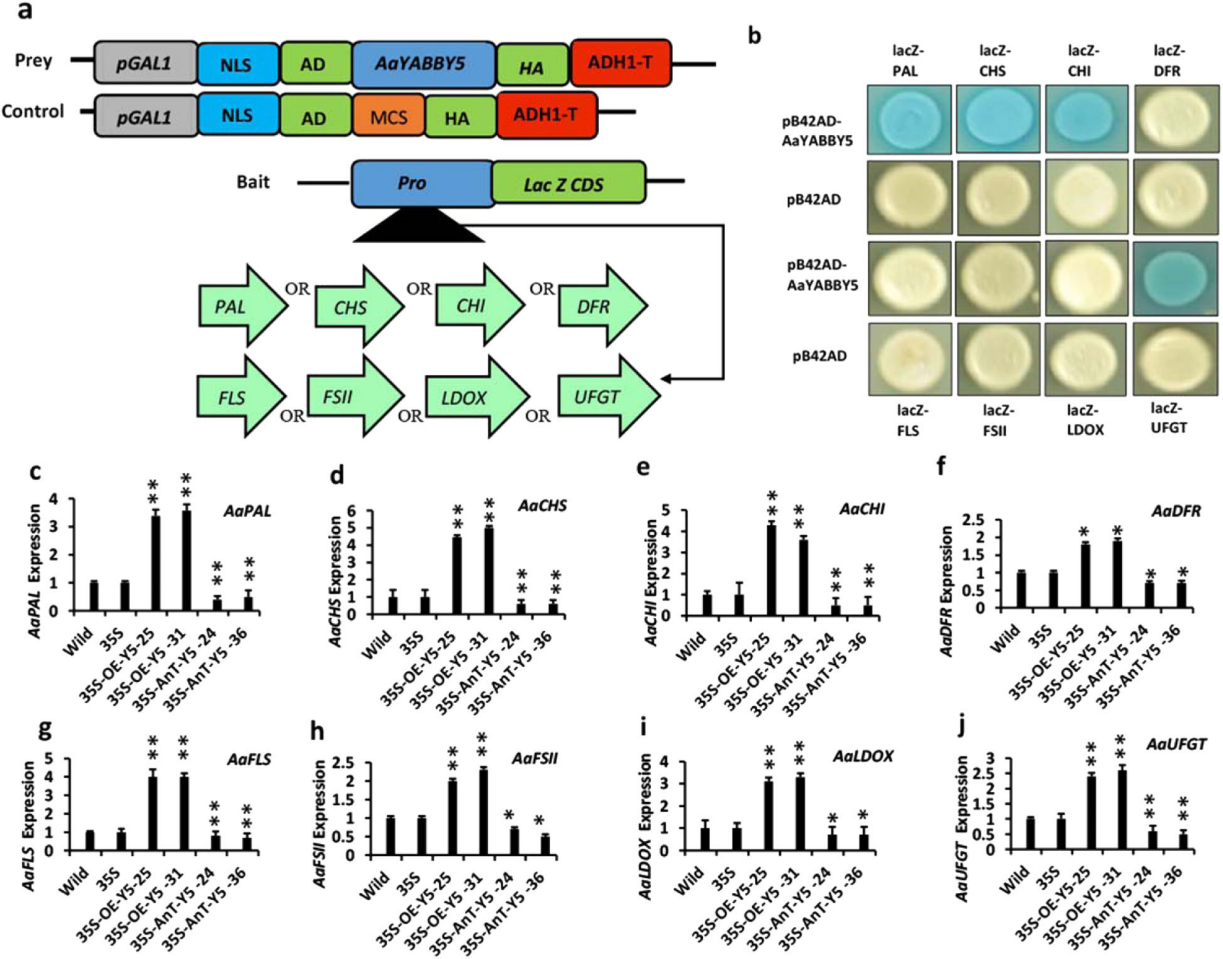
Yeast one-hybrid assay (Y1H) and real-time PCR analysis of flavonoid biosynthetic genes in AaYABBY5-OE plants, AaYABBY5 AnT. plants, pHB vector-containing (35S) plants, and wild-type *A. annua* plants. **a** Sketch map of prey and bait constructs used to perform the Y1H assay. The coding sequence of the prey protein AaYABBY5 was cloned into the pB42AD vector under the *GAL1* inducible promoter sequence as a fusion to the NLS; nuclear localization sequence, AD; activation domain, and HA (hemagglutinin) epitope tag, whereas promoters of *PAL, CHS, CHI, DFR, FLS, FSII, LDOX*, and *UFGT* were cloned into the placZ2µ vector as fusions with the *lacZ* reporter gene to form the bait strains. **b** AaYABBY5 directly bound to full-length *PAL, CHS, CHI*, and *UFGT* promoters in yeast cells cotransformed with these bait strains along with pB42AD-AaYABBY5, as shown by the blue-colored phenotype of yeast clones, but not with empty pB42AD. *FLS, FSII*, and *LDOX* did not show positive results. **c**–**j** show the relative expression of the *PAL, CHS, CHI, DFR, FLS, FSII, LDOX*, and *UFGT* genes in selected *AaYABBY5* OE, *AaYABBY5* AnT, 35S, and wild-type (W) *A. annua* plants. Gene expression was found to increase in *AaYABBY5*-OE plants, whereas in *AaYABBY5* AnT. plants, a decrease in the expression of *PAL, CHS, CHI, DFR, FLS, FSII, LDOX*, and *UFGT* was found. β-Actin was used as an internal control. The graph shows the mean values ± SD of three experimental replicates. Error bars show the standard deviation for the sample, *n* = 3. Statistical significance was determined using the Student’s *t* test. **P < 0.01, *P < 0.05

Transactivation assays using *N. benthamiana* revealed that AaYABBY5 mediated a significant increase in the activities of *PAL, CHS, CHI*, and *UFGT* promoters in vivo. Therefore, to determine the molecular basis of this regulation and whether AaYABBY5 directly activates them, a Y1H assay was performed. The experiment demonstrated the binding of the pB42AD-AaYABBY5 fusion protein (blue color appearance), but not pB42AD alone (no color), to the *PAL, CHS, CHI*, and *UFGT* promoter sequences, indicated by the activation of the *lacZ* reporter gene, which produces ß-galactosidase and cleaves the X-gal present in growth medium to a compound with a blue-colored phenotype. The experiment was repeated three times to validate the results. No colored phenotype was found for the *DFR, FLS, FSII*, and *LDOX* promoters (Fig. 4b).

The results of the Y1H assay were consistent with the findings of the dual-luciferase reporter assay. In other plants, including *A. thaliana*, flavonoid genes, and their transcriptional regulation are well-studied. The *A. thaliana FIL* gene has been previously reported to be a positive regulator of anthocyanins by activating the *MYB75* gene^42^. To our knowledge, for the first time, we found the molecular basis of the transcriptional regulation of flavonoid biosynthetic pathway genes, *PAL, CHS, CHI*, and *UFGT* in *A. annua*. These results indicated that AaYABBY5 might have the potential to regulate both flavonoid and anthocyanin biosynthesis in *A. annua*. Therefore, these contents were measured and compared in *AaYABBY5* OE and *AaYABBY5* AnT. *A. annua* plants.

### AaYABBY5-overexpressing *A. annua* plants showed a consistent significant increase in the expression of genes from *AaPAL* to *AaUFGT*

After analyzing the binding of AaYABBY5 to the promoters of early (flavanone) and late (anthocyanin) flavonoid biosynthetic genes, it was important to study its functions in *A. annua*. As expected, the gene expression analysis revealed an increase in the expression of all genes under study. The comparative expression of these genes indicated that *AaCHS* showed higher expression than all other genes under study. Similarly, the expression of *DFR*, *FSII*, and *UFGT* was lower than that of *PAL, CHS, CHI, FLS*, and *LDOX* (Fig. 4c–j).

Although *DFR, FLS, FSII*, and *LDOX* were not activated by AaYABBY5, a higher expression of these genes in *AaYABBY5*-OE plants was found. It is proposed that the increased flux provided by *PAL, CHS*, and *CHI* activates downstream pathway enzymes by increasing the concentration of substrates for the enzymes acting downstream: *DFR, FLS, FSII*, and *LDOX*. *AaYABBY5* overexpression not only increased the expression of its direct target genes but also the flavonoid pathway under study.

### AaYABBY5 antisense *A. annua* plants showed a significant decrease in the expression of flavonoid biosynthetic genes

The AaYABBY5 protein activates flavonoid biosynthetic genes, and the increased expression of AaYABBY5 led to a dramatic increase in the expression of flavonoid-regulating genes. Transcript levels of *PAL, CHI, CHS, DFR, FLS, FSII, LDOX*, and *UFGT* were analyzed in *AaYABBY5* antisense RNA-containing plants. As expected, a significant decrease in the expression of the genes under study was found in *AaYABBY5* AnT. plants (Fig. 4c–j). Overall, from these findings, a clear understanding of AaYABBY5-regulated flavonoid biosynthesis was obtained. To validate the above findings, flavonoid and anthocyanin concentrations were measured and compared among *AaYABBY5* OE, *AaYABBY5* AnT, wild-type/control plants, and vector-containing plants.

### AaYABBY5 positively regulates flavonoid biosynthesis

**Fig. 5:**
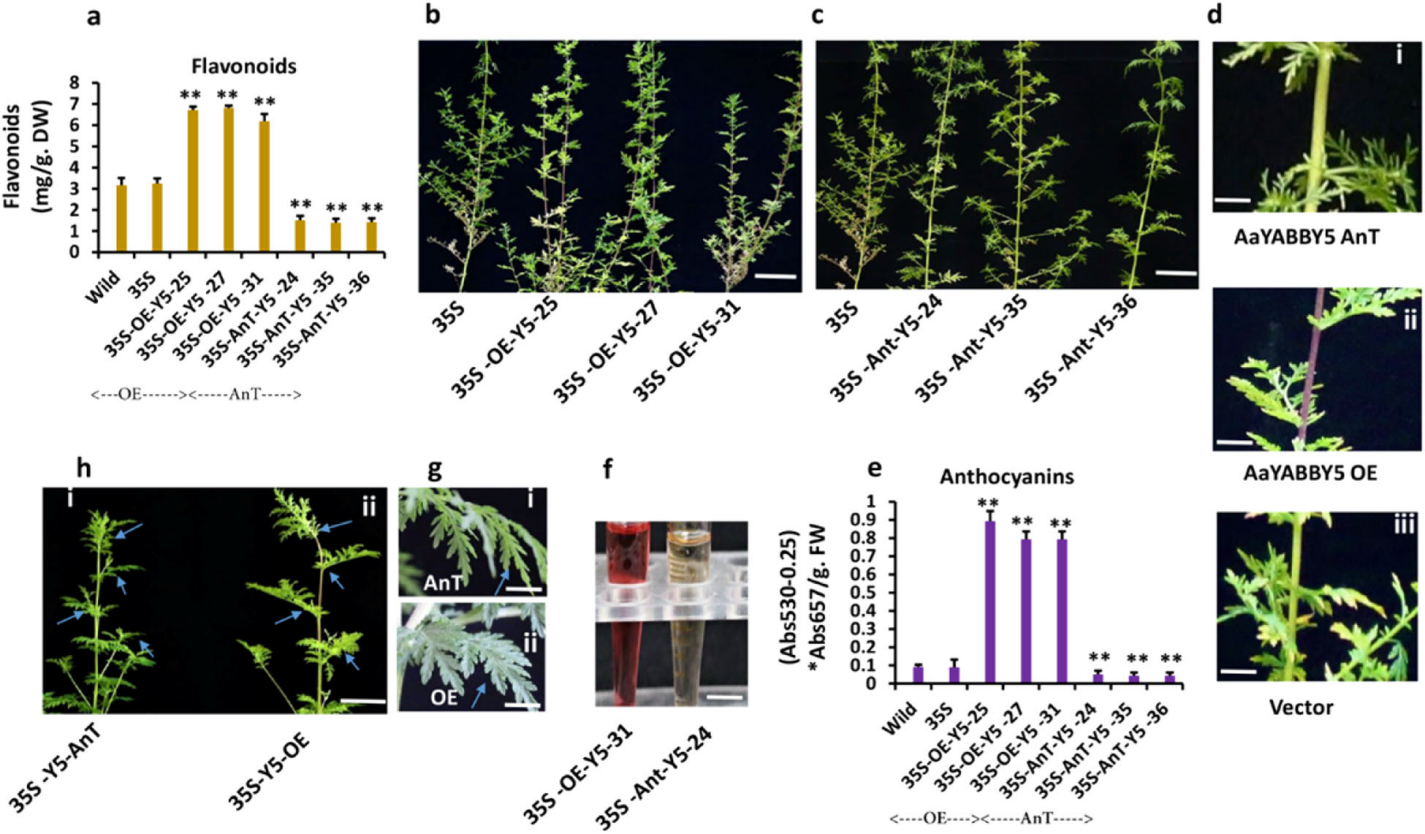
Comparative analysis of flavonoid and anthocyanin contents in AaYABBY5-OE plants, AaYABBY5 AnT. plants, and control plants, as well as phenotypic analysis of transgenic plants. **a** Comparative analysis of total flavonoid content in *AaYABBY5*-OE plants, *AaYABBY5* AnT. plants, vector-containing (35S), and wild-type plants, showing a significant increase in the total flavonoid content of *AaYABBY5* OE plants compared to *AaYABBY5* AnT. plants and control ones. Similarly, AnT. plants showed a decrease in the concentration of flavonoids. **b**–**d** Phenotypic analysis of transgenic plants showing deep purple pigmentation in the stems of *AaYABBY5*-OE plants, whereas no purple-color phenotype was found in wild-type and/or antisense *AaYABBY5* plants. Scale bars = 8 cm in (**b**) and (**c**) and 4 cm in (**d**). **e** Comparative analysis of anthocyanin concentrations in *AaYABBY5*-OE plants, *AaYABBY5* AnT. plants, and vector-containing (35S) and wild-type plants, showing a significant increase in the total anthocyanin content of *AaYABBY5*-OE plants compared to *AaYABBY5* AnT. plants and control ones. In contrast, *AaYABBY5* AnT. plants showed a decrease in the concentration of anthocyanins. **f** Coloric representation of anthocyanin enrichment in the anthocyanin extract of *AaYABBY5*-OE plants showing the red color of the anthocyanin extract, an indication of an increased amount of anthocyanins. Scale bars = 4 cm. **g**, **h** Phenotypic analysis showing broader lamina in *AaYABBY5*-OE plants (ii) compared to *AaYABBY5* AnT. plants (i). Scale bars = 3.5 cm and 10 cm in **g** and **h**, respectively. The graph shows the mean values ± SD of three experimental replicates. Error bars show standard deviation for sample, *n* = 3. Statistical significance was determined using Student’s *t* test. **P < 0.01, *P < 0.05

Flavonoids are polyphenolic plant secondary metabolites that are classified into different types. In this study, we found that AaYABBY5 positively regulates the *PAL, CHI, CHS*, and *UFGT* genes. The results of real-time PCR also verified the increased expression of these genes in *AaYABBY5* overexpression plants. These findings revealed a positive behavior of AaYABBY5 toward flavonoid biosynthesis in *A. annua*. To justify this, flavonoid contents from *AaYABBY5* overexpression plants, *AaYABBY5* antisense plants, and control plants were measured using the aluminum chloride (AlCl3) colorimetric method with quercetin as a standard. As expected, the results revealed an increased concentration of flavonoids in *AaYABBY5* overexpression plants compared to *AaYABBY5* antisense or control plants (Fig. 5a), proving the function of AaYABBY5 as a positive regulator of flavonoid biosynthesis.

### AaYABBY5 promotes anthocyanin biosynthesis, displaying a purple phenotype in *A. annua* stems

Anthocyanins are secondary metabolites widely present in plant species and are responsible for the purple, bluish, and pinkish pigmentation of different plant parts. In this study, we observed deep purple pigmentation in the stems of *AaYABBY5* overexpression plants, whereas *AaYABBY5* antisense plants and control plants showed no purple phenotype (Fig. 5b, c).

To test whether *AaYABBY5* is involved in the regulation of anthocyanin biosynthesis in *A. annua*, we measured and compared the total anthocyanin content of stem extracts of control plants and pHB/wild-type, *AaYABBY5*-overexpressing, and *AaYABBY5* antisense plants. It is known that anthocyanins produce a red color when treated with acids. Consistent with this, the anthocyanin extracts from purple-colored *AaYABBY5*-OE plants were red-colored in acidic medium (Fig. 5f). As expected, a significant increase in the concentration of anthocyanins was found in the stems of plants overexpressing *AaYABBY5* compared to the control and *AaYABBY5* antisense plants (Fig. 5e). Previous investigations have demonstrated that the YABBY family TF *AtFIL* is a positive regulator of anthocyanin biosynthesis in the model plant *A. thaliana* through activating the *MYB75* promoter; however, no direct link to the genes regulating anthocyanins or flavonoids was reported in *A. annua*^42^. *MYC2* has been reported to be responsible for an increase in the anthocyanin content, giving a purple phenotype to AaMYC2-overexpressing stems^28^; however, the mechanism underlying this regulation was not studied. To our knowledge, we provide the first molecular basis of flavonoids, including anthocyanin regulation by the YABBY family transcription factor AaYABBY5. It was supposed that AaYABBY5 activates anthocyanin biosynthesis through the direct activation of *UFGT* and upstream pathway genes.

### *AaYABBY5* overexpression results in broader leaf lamina and increased trichome density

The primary function of YABBY family transcription factors found in seed plants is the control of leaf development, increasing the size of the leaf lamina, and maintaining organ polarity^50^; therefore, it was important to determine whether *AaYABBY5* overexpression and/or its downregulation in *A. annua* affected leaf morphology. Phenotypic analysis of *AaYABBY5*-OE plants, *AaYABBY5* AnT. plants, and control plants showed that the leaves of *AaYABBY5*-OE plants have broader leaf lamina, whereas in *AaYABBY5* AnT. plants, the leaf lamia was reduced. Leaves of *AaYABBY5* AnT. plants were radialized compared to that of OE plants and/control plants (Fig. 5g, h). Trichomes are the sites of secondary metabolite synthesis, and studies have reported that flavonoids are synthesized in trichomes^47^.

**Fig. 6:**
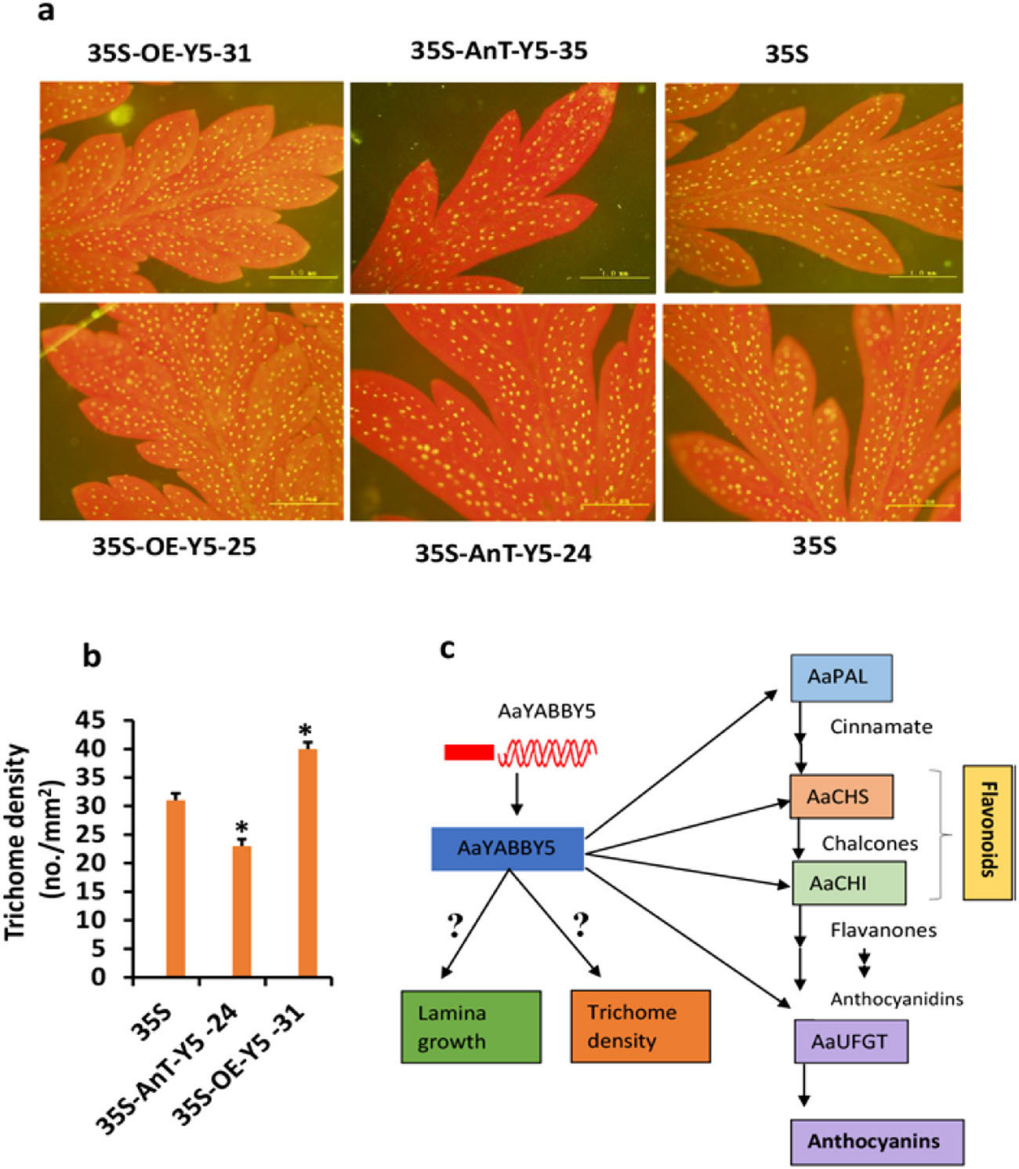
Measurement of glandular trichome density and working model of regulation of flavonoid biosynthesis by AaYABBY5. **a** Trichome densities on the leaf surface of transformed and control plants were calculated, and images were captured using fluorescence microscopy (Olympus, Japan). The trichome densities of *AaYABBY5*-OE plants were higher than those of the control and *AaYABBY5* AnT plants. Scale bars represent 200 µm. **b** Graphical representation of trichome densities of control, *AaYABBY5* AnT., and *AaYABBY5*-OE plants calculated as numbers per millimeter square (no./mm^2^). Data = mean values ± standard deviation for *n* = 4. Error bars represent standard deviation for *n* = 4. Student’s *t* test with paired and two-tailed distribution methods. * represents *P* < 0.05. **c** The AaYABBY5 protein directly targets the promoters of *PAL*, lying upstream of the flavonoid biosynthetic pathway. The *PAL* gene, once activated, provides increased metabolic flux toward the first committed step of flavonoid biosynthesis. AaYABBY5 also directly binds to and activates the *CHS* and *CHI* promoters, providing increasing concentrations of flavonones (total flavonoid content). Going through multistep reactions, in the final step, anthocyanidins, which are the precursors of anthocyanins, are converted into anthocyanins by AaYABBY5 through the direct activation of the *AaUFGT* promoter. AaYABBY5 also regulates trichome number and leaf lamina growth through an unknown mechanism

Trichome development is a part of leaf development. Knowing that AaYABBY5 regulates leaf lamina, the trichome densities on the leaf surfaces were compared among transgenic plants. The trichome densities on leaf surfaces from *AaYABBY5* overexpression plants, *AaYABBY5* antisense plants, and control plants with an empty vector were calculated. A significant increase in the trichome densities of *AaYABBY5* overexpression plants was found compared to control plants, whereas for *AaYABBY5* antisense plants, trichome density was found to significantly decrease. These results show that AaYABBY5 is also a positive regulator of trichome development (Fig. 6a, b).

AaHD1 (homeodomain protein 1) is involved in the initiation of both glandular and nonglandular trichomes in *A. annua*.^49^; however, no protein interactions between AaYABBY5 and AaHD1 were found. It is proposed that AaYABBY5 might activate the promoter of *AaHD1*, which regulates trichome development. This research opened paths for future research where the molecular mechanism of the regulation of trichome development by AaYABBY5 can be found.

## Discussion

*Artemisia annua* is a renowned traditional Chinese medicinal plant that has been used in China for a long time as a treatment of fever, inflammation, and malaria, and its use now extends to Europe and North America^50^. *A. annua* is rich in hydroxylated flavonoids and polymethoxylated flavonoids with useful biological activities, such as antioxidant, anticancer, and antimalarial activities^51^. The most interesting feature of *A. annua* flavonoids found recently is to synergize antimalarial and anticancer compounds, most importantly artemisinin^3,50^. Despite the worthwhile effects of flavonoids, research on the molecular and transcriptional regulation of flavonoid biosynthesis in *A. annua* is limited. Recently, chalcone isomerase (*AaCHI*) phenylalanine ammonia lyase (*AaPAL1*), flavanone-3-hydroxylase (*AaF3*′*H*), and *AaFLS1* have been cloned and characterized as enzymes that take part in flavonoid biosynthesis^16,17,26,27^. A few transcription factors from the bHLH and MYB families have been recently reported as regulators of flavonoids in *A. annua*. Examples include the overexpression of *MYC2* (which binds to G boxes present in the *AaCYP71AV1* and *AaDBR2* promoters) in *A. annua*, resulting in an increased level of both anthocyanins and artemisinin^28^. Mostly, R2R3-MYB has been shown to play regulatory roles in flavonoid biosynthesis in *A. annua*. Recently, the MYB family transcription factor AaTAR2 from *A. annua* was reported to synergistically regulate both artemisinin and flavonoids^29^; however, the molecular mechanism regulating flavonoid production was not elucidated.

Arabidopsis FIL activates *MYB28*, which activates aliphatic glucosinolate biosynthetic genes^43^. *AtYAB3* is a positive regulator of MYB75, an activator of anthocyanin biosynthesis^42^. MsYABBY5 is a direct activator of WRKY75, which possibly represses terpene biosynthesis in *M. spicata*^44^. OsYABBY4 is a negative regulator of the gibberellin (GA) 20-oxidase 2 gene (*GA20ox2*), and SLR1 is involved in gibberellic acid responses in rice^52^. Because of the functions performed by YABBY genes in other plants and their role in anthocyanin biosynthesis in *A. thaliana*, it was important to determine the role of YABBY TFs in flavonoid biosynthesis. The role of YABBY family transcription factors in flavonoid biosynthesis in *A. annua* was not previously characterized. This study provides AaYABBY5 as a novel regulator of flavonoid biosynthesis in *A. annua*.

We screened the promoters of *PAL* (from upstream precursors of the flavonoids pathway), *CHS* (involved in the first committed step toward artemisinin biosynthesis), *CHI*, *DFR* (acting downstream of *CHS*) *FLS* and *FSII* as flavonols and flavones regulators, respectively, and *LDOX* and *UFGT* (which are responsible for anthocyanin and pigment formation), using dual-luciferase reporter assay and Y1H assay, and found that *PAL, CHS, CHI*, and *UFGT* are directly regulated by AaYABBY5 (Figs. 3 and 4b). The direct binding of AaYABBY5 to these promoter regions provided the molecular basis for the regulation of flavonoids in *A. annua*. The results of the Y1H assay were consistent with the results of the dual-Luc assay.

In *A. thaliana*, AtFIL was reported to be a target of JAZ3 and a regulator of anthocyanin biosynthesis by activating the promoter of *MYB75* (*PAP1*); however, no direct interaction with flavonoid pathway genes was reported^42^. In plants, including *A. thaliana*, MYB family proteins have been proven to be candidate genes that regulate flavonoid biosynthesis. AtMYB12 has been found to be a regulator of *CHS* and *FLS* genes through Myb-recognition elements (MREs) present in the promoters of these genes^53^. The R2R3-MYB transcription factor PAP1 displayed a purple phenotype in transgenic Arabidopsis by upregulating various genes involved in anthocyanin biosynthesis^54^. Recently, the MYB family transcription factor AaTAR2 was found to synergistically regulate both artemisinin (indirectly) and flavonoids in *A. annua;* however, the molecular mechanism regulating flavonoid production was not clearly elucidated, and no direct interaction with the artemisinin biosynthetic pathway was given^29^. Based on these findings, it was important to study the regulation of flavonoid biosynthetic pathway genes in *AaYABBY5* overexpression plants, *AaYABBY5* antisense plants, and control plants.

To confirm the role of AaYABBY5 in the regulation of flavonoid biosynthesis, comparative expression and biochemical analysis of transgenic *A. annua* containing pHB-35S: AaYABBY5, pHB-35S: anti-AaYABBY5, pHB, and wild-type plants revealed a significant upregulation of the *AaPAL*, *AaCHI*, *AaCHS*, *AaFLS, AaFSII, AaLDOX*, and *AaUFGT* genes under study (Fig. 4c–j). We propose that AaYABBY5 binds to the *PAL* promoter and activates *PAL* gene expression, which provides metabolic flux to the flavonoid pathway. The overexpression of *AaYABBY5* in *A. annua* provides elevated levels of the AaYABBY5 protein, which binds to and activates the *PAL* gene. The increased activity of PAL converts more phenylalanine molecules to cinnamate, which provides precursor molecules for entry into the flavonoid pathway. This provides an increase in the concentration of the substrate molecules for the CHS enzyme. Knowing that *AaCHS* catalyzes the first committed step of the flavonoid pathway (Fig. 1) and considering our findings that AaYABBY5 has a strong binding capability to the *AaCHS* promoter, it is supposed that increased *AaCHS* expression accelerates the biosynthesis of chalcones, which provides flux to downstream reactions, providing an increase in the expression of genes lying downstream of *AaCHS* in the flavonoid biosynthesis in *AaYABBY5*-OE *A. annua* plants. When *PAL* and *CHS* are activated, they perform their function and trigger the activation of downstream reactions, providing accelerated substrate recovery, as a result of which more enzyme molecules are activated, as found by increases in the transcript levels of *CHI, FLS, FSII, LDOX*, and *UFGT*. Among these, *CHI* and *UFGT* are also activated directly by AaYABBY5. Therefore, it is concluded that the increase in the concentration of flavonoids in OE *AaYABBY5* transgenic *A. annua* is governed by AaYABBY5, which directly activates the *PAL, CHS*, and *CHI* genes. The results were further validated by analyzing *A. annua* plants with suppressed *AaYABBY5*, wherein the expression of genes regulating flavonoid biosynthesis and flavonoid concentration were found to be significantly decreased (Figs. 4c–j and 5a).

In *A. annua*, chalcone isomerase (*AaCHI*), phenylalanine ammonia lyase (*AaPAL1*), flavanone-3-hydroxylase (*AaF3*′*H*), and *AaFLS1* have been characterized as enzymes that take part in the flavonoid biosynthesis pathway^16,17,26,27^. The results obtained in this study are in accordance with these previous findings that, when the expression of *PAL* or *CHI* is increased, it thereby enhances the flavonoid concentration. The term flavonoids used in this study indicates the concentration of flavones and flavonols according to the principle of the AlCl3 method. Kaempferol and quercetin, are important flavonoids of *A. annua* that belong to the flavonol group, and a significant increase in the flavonoid concentration in *AaYABBY5*-OE plants might correspond to the enhanced accumulation of kaemferol and quercetin, which favors the synergistic role of AaYABBY5 toward the regulation of flavonoids (present study) and artemisinin biosynthesis^48^.

Anthocyanins are a group of flavonoids that are synthesized downstream of the *AaDFR* gene, governed by *AaLDOX* and *AaUFGT* in two-step reactions. Physiological analysis of transgenic plants and control plants revealed the presence of purple pigmentation in the stems and petioles of *AaYABBY5*-OE plants, whereas no colored phenotype was observed in the control and *AaYABBY5* AnT. plants (Fig. 5b, c). Gene expression analysis also revealed a significant increase in the expression of *AaDFR*, *AaLDOX*, and *AaUFGT*, as well as an increase in the concentration of anthocyanins in *AaYABBY5*-OE plants. As expected, the transcript levels of *AaDFR, AaLDOX*, and *AaUFGT* and the resulting anthocyanin concentrations were significantly decreased in *AaYABBY5* AnT. plants (Fig. 4f, i, j). AaYABBY5 cannot directly bind to *DFR* and *LDOX* promoters. It is supposed that the activation of early flavonoid genes, *CHI* and *CHS*, results in the activation of later steps, which enables the rapid accumulation of anthocyanin precursor molecules; pelargonidin, delphinidin, and cyanidin are collectively named anthocyanidins. Precursor anthocyanins called anthocyanidins are glycosylated by the enzyme UDP-glucose flavonoid 3-*O*-glucosyl transferase (UFGT) to produce colorful and stable compounds called anthocyanins^25^. We hypothesized that the increase in the concentration of anthocyanidins, which are substrate molecules for the UFGT enzyme, and direct activation of *AaUFGT* by AaYABBY5 increases the rate of forward reaction toward anthocyanin biosynthesis governed by glucosylation. These findings prove that AaYABBY5 is a positive regulator of anthocyanins.

Regarding the basic role of YABBY genes is in the regulation of leaf development, in this study, comparative phenotypic analysis of *AaYABBY5*-OE, *AaYABBY5*-AnT., and control plants showed that as expected, the leaf lamina was broader in overexpression plants (Fig. 5g–h (ii)), whereas radialized lamina was observed in antisense plants (Fig. 5g–h (i)). The differences can also be seen in Fig. 5d (i–iii). These results showed that AaYABBY5, in addition to regulating secondary metabolite biosynthesis, also regulates primary metabolism associated with leaf growth. Considering these results, trichomes, which are the sites of secondary metabolite synthesis in plants, were studied. Comparative trichome densities of *AaYABBY5*-OE, *AaYABBY5*-AnT., and control plants revealed higher trichome densities in *AaYABBY5*-OE plants than in the control plants and *AaYABBY5*-AnT. plants; however, the molecular mechanism of its regulation is the target of future research (Fig. 6a, b). A model representing the functions of AaYABBY5 found in this study is given in Fig. 6c.

This study, for the first time, provided transcriptional regulation of flavonoids using YABBY family transcription factors, and this is the first report on the direct transcriptional regulation of flavonoid pathway genes in *A. annua*. Our previous data that AaYABBY5 directly activates artemisinin biosynthetic pathway genes^48^ and our present findings regarding AaYABBY5-mediated direct regulation of flavonoids, including anthocyanins, provide evidence of the parallel transcriptional regulation of artemisinin and flavonoid biosynthesis by AaYABBY5 in *A. annua*, thus proving that AaYABBY5 can be a good candidate gene to provide increasing concentrations of the two biologicals at the same time in *A. annua*.

## Materials and methods

### Cloning of promoters

The *A. annua* genomic assembly data present in NCBI were screened to find putative promoter sequences upstream of the initiation codon ATG using ORF sequences of *AaPAL, AaCHS*, *AaCHI*, *AaFLS, AaFSII*, *AaDFR*, *AaLDOX*, and *AaUFGT* as queries.

Approximately 2 kb *PAL* promoter, 1.6 kb *AaCHS* promoter, 1.4 kb *AaCHI* promoter, 1.4 kb *AaFLS* promoter, 1.2 kb *AaFSII* promoter, 1.2 kb *AaDFR* promoter, 1.9 kb *AaLDOX* promoter, and 1.7 kb promoter sequence of *AaUFGT* were cloned. Genomic DNA extracted from young leaves of *A. annua* was used as a template, and amplification was performed using a KOD Plus PCR kit (Toyobo). DNA bands purified using the DNA Gel Extraction Kit (AxyPrep) were ligated to the PLB simple vector (Tiangen Biotech, China) containing carbenicillin-resistance genes according to the manufacturer’s instructions provided with the Lethal Based Fast Cloning Kit (Tiangen), followed by transformation into DH5α-competent cells (Invitrogen).

### Bioinformatic analysis of promoters of flavonoid biosynthetic genes

The cloned promoter sequences were confirmed by Sanger sequencing and analyzed for the prediction of putative YABBY-binding sites^37^ using PLANTPAN3.0.

### Identification of AaYABBY5 as a potential flavonoid-regulating transcription factor

The transcriptomic data of six different tissues of *A. annua* generated by our research center were screened, and reads of each YABBY gene, flavonoid biosynthetic genes, and artemisinin biosynthetic genes in six different tissues (trichome, bud, stem, root, leaf, and seed) were obtained using a BLASTN search against each database with an *E* value <1 × 10^–6^, and read counts were normalized by calculating the value of reads per kilobase per million (RPKM). To predict the YABBY transcription factor, which may regulate flavonoid-regulating genes, coexpression analysis was performed on the basis of RNA sequencing data using Multi-Experiment Viewer (MeV4.9.0) software^55^.

### Plant material and growth conditions

The seeds of the *A. annua* cultivar Huhao #1 used in this study were collected from our previous stably transformed transgenic *A. annua* plants with overexpressed AaYBABBY5 (35S—AaYABBY5), silenced AaYABBY5 (35S-anti-AaYABBY5), empty vector-containing plants (35S), and wild-type or control plants (untransformed). Details about construct preparation and *A. annua* transformation are given in ref. ^48^. *A. annua* plants (transgenic and control) were also propagated from stem cuttings. For such cutting propagations, 8–10-cm long stems or side shoots were cut just below a leaf and propagated in plant trays supplemented with soil matrix. After extensive rooting development and proper growth as a complete plant, the cuttings were propagated to pots. In the transient agroinfiltration assay, *N. benthamiana* plants with young leaves and expanded lamina were used. Both plant types were grown under the same conditions of temperature: 25 °C ± 2 °C and photoperiod: 16 h light:8 h dark.

### Transient *N. benthamiana* infiltration system

To perform a transient infiltration assay in the *N. benthamiana* system using a dual-LUC kit, effector and reporter strains were prepared as follows. For the effector strain, the open-reading frame of *AaYABBY5* without its terminal codon was amplified using primer sequences specifically designed for cloning into the pENTR-TOPO vector, followed by subsequent recombination into the Gateway destination vector pEarleyGate 104-YFP (N-terminal YFP) (Invitrogen, USA). For the reporter construct, promoter sequences of *PAL*, *CHS, CHI, FLS*, *FSII*, *LDOX*, and *UFGT* were amplified with primer sequences containing adapter sequences specific to the pGreenII 0800-LUC plasmid. The purified fragment was subsequently ligated into the pGreenII 0800-LUC plasmid through HindIII and PstI sites to generate pro:*LUC* constructs, according to a previous protocol^48^. The plasmids were transformed into *A. tumefaciens* GV3101 competent cells with pSoup-p19 to help suppress gene silencing. The process of agrobacterium culture preparation, infiltration into the *N. benthamiana* leaf tissues, sample collection, and preparation for the measurement of LUC activity was followed according to a previous protocol^48^.

### Yeast one-hybrid system

For the Y1H assay, bait and prey constructs were prepared as follows. The promoter regions of *AaPAL, AaCHS*, *AaCHI*, *AaFLS, AaFSII*, *AaDFR*, *AaLDOX*, and *AaUFGT* were amplified using primer sequences with 5’ adapter sequences specific to the placZ vector and subsequently ligated into placZ using one-step cloning, following the protocol given by ClonExpress II (Vazyme). Similarly, the open-reading frame of *AaYABBY5* with its terminal codon was amplified using primer sequences with 5’ adapter sequences specific to the pB42AD vector (Addgene) and subsequently ligated into it. Both constructs were confirmed by sequencing. Promoter sequences cloned in placZ were used as bait against pB42AD- AaYABBY5 prey.

The assay was performed according to the manual of the Matchmaker Gold Y1H system given in the *Yeast Protocols Handbook*, Clontech, (Japan). The combination of pB42AD-*AaYABBY5* with the respective promoter sequence in placZ was cotransformed into EGY48 competent cells. The combination of negative controls pB42AD- *AaYABBY5* with empty lacZ (lacZ-0), pB42AD-0 with the placZ promoter, and empty pB42AD (pB42AD-0) with empty placZ (placZ-0) was also transformed into EGY48 cells. Transformed yeast cells were streaked on SD-Tryptophan-Uracil (SD/-Trp-Ura). After 3 days, colonies were shifted to SD/-Trp-Ura supplemented with X-Gal (5-bromo-4-chloro-3-indolyl-ß-D-galactopyranoside). The experiment was repeated three times.

### RNA extraction and gene expression analysis

To check the expression pattern using real-time PCR, RNA extraction and cDNA synthesis were performed using an RNAprep Pure Plant Kit (Tiangen) and PrimeScript RT Reagents (Takara), respectively. Pure and good quality 500 ng RNA was used as a template for cDNA synthesis using reverse transcriptase enzyme. The cDNA was diluted in RNase-free ddH2O at a 1:40 ratio. Six microliters of diluted cDNA was used as a template for real-time PCR analysis using the protocol of ref. ^28^ with minor modifications.

SYBR Green Super real premix was used to prepare a master mix, and a Roche Light Cycler 96-well Real-Time PCR Machine (Roche, Switzerland) was used to quantify the expression. The PCR program was set up at 40 cycles, each containing 2 min at 95 °C for initial denaturation, 20 s at 95 °C for denaturation, 20 s at 54 °C for annealing, and 20 s at 72 °C for the extension. β-Actin was used as a standard. For each experimental and control sample, three repeats were used.

### Preparation of flavonoid extracts and measurement of total flavonoid content

For quantitative determination of the total flavonoid content, leaf samples from each plant were collected and ground to powder form using liquid nitrogen (−70). Approximately 1 g of each leaf sample was placed in 50-ml glass flasks and mixed with 5 ml methanolic solution (70%). The extraction was performed twice using an ultrasonic processor (DL-720B) at a frequency of 55 Hz and a temperature of 30 °C for 35 min. The supernatants containing extracts were collected following centrifugation at 6000 rpm for 10 min. Approximately 3 ml of each extract was filtered through a 0.22-μm nitrocellulose filter and transferred to glass vials.

The total flavonoid content of *A. annua* exudates was calculated using the aluminum chloride (AlCl3) colorimetric method according to protocol^56^. The working principle of the AlCl3 colorimetric method is that AlCl3 forms acid-stable complexes with the C-4 keto group and the C-3 or C-5 hydroxyl group of flavones and flavonols. It also forms complexes with the orthodihydroxyl groups in the A- or B-ring of flavonoids. For sample preparation, 10 μL of each extract was diluted in 150 ml of deionized water, followed by the addition of 20 μl potassium acetate (1 M) and 20 μl of aluminum chloride (10%). The final volume of the 200 μl reaction mixture was incubated at 37 °C for 30 min. The absorbance was measured at 415 nm. The total flavonoid content was calculated as quercetin equivalents. The absorbance of three independent biological repeats from each plant type: *AaYABBY5* OE, *AaYABBY5* AnT., as well as control plants, was measured in triplicate. Error bars indicate the SD of the average flavonoid concentration.

### Preparation of anthocyanin extracts and measurement of anthocyanins

To extract anthocyanins/pigments from the stem tissues of *A. annua* plants, the following protocol^57^ was used with minor modifications. Samples from each plant were collected and ground in liquid nitrogen to powder form. A 1 g sample was mixed with 10 ml of acidic methanol (70% methanol with 0.1% HCl) and processed by an ultrasonic processor (DL-720B) at 50 Hz for 40 min. The processed samples were kept at 4 °C overnight mixing and incubation under dark conditions.

The extracts were purified by centrifugation at 12000 rpm for 10 min by filtration. The supernatants were separated and filtered through 0.22-μm nitrocellulose filters. Absorbance at 530 nm and 657 nm was measured using a microplate reader (BioTek, ELx 800). Relative anthocyanin content was found by (A530–0.25×A657)/FW (FW is the fresh weight of sample in grams). The absorbance of each sample was measured in triplicate. Three independent biological replicates from each plant type were used. Error bars indicate the standard deviation of the average anthocyanin concentration.

### Measuring trichome density

To calculate the trichome densities, mature and healthy leaves from three-month-old *A. annua* plants were used. To ensure comparison at the same growth level, the 8th leaf below the meristem was selected from transformed and control plants. Images were captured using fluorescence microscopy (Olympus, Japan) under the following conditions: 5x magnification and 450–480 nm excitation wavelength. The images were analyzed by using ImageJ 1.51k software to measure the leaf area and calculate trichome numbers. Finally, trichome densities were calculated from the ratio of trichome number to the leaf area. The experiment was repeated three times, using the 8th leaf from three different plants of each line, to obtain statistically significant results.

### Primer sequences

The primer sequences used in this study were prepared using Primer 3 software and are given in Supplementary Table S1.

### Statistical analysis

Statistical analysis was performed using Student’s *t* test with paired and two-tailed distribution methods. ** and * represent statistically significant group differences for *P* < 0.01 and <0.05, respectively.

## Supplementary information

Supplementary material

## Data Availability

All data supporting this study are included in the article and its supplementary files.
