# Supplementary material for: Transcriptional regulation of flavonoid biosynthesis in Artemisia annua by AaYABBY5
Source: Hortic Res. 2021 Dec 1;8:257. doi: 10.1038/s41438-021-00693-x (PMC8632904; doi:10.1038/s41438-021-00693-x)
Supplement: Supplementary file 1 — Supplementary material [file 41438_2021_693_MOESM1_ESM.doc]

**Supplementary data**

Supplementary data includes table S1 of primer sequences.

**Table S1**

Primer sequences used in the experiments

| **Primer** | **Purpose** | **Sequence (5'→3')** |
| --- | --- | --- |
| PLB-PAL-PF | Cloning | 5’TCAGTAGGCCACGGATTGGA3’ |
| PLB-PAL-PR | Cloning | 5’TTGGTGCAAAAGACAATGAGCA3’ |
| AaYABBY5F | Cloning | 5' TAAAGAGAGACTAACTTGCTTGG 3' |
| AaYABBY5R | Cloning | 5' ATAAGAGGATTTGCTTTAGATTTC 3' |
| PLB-CHI-PF | Cloning | 5'AGTCAGTGTGCCAATGGTGT3' |
| PLB-CHI-PR | Cloning | 5'GAAAAATACCTGCGCCACCG3' |
| PLB-CHS-PF | Cloning | 5'CTCGAGACCTGAGCGTTAGA3' |
| PLB-CHS-PR | Cloning | 5'CTGGGAGCCGGATTTCACTA3' |
| PLB-DFR-PF | Cloning | 5'TGGTTTGTGTGACAGGTGCT3' |
| PLB-DFR-PR | Cloning | 5'GGTCAATGGTCGGGTAGAGC3' |
| PLB-FLS-PF | Cloning | 5'ATGGTGACTGGTATGATGTTGCTTA3' |
| PLB-FLS-PR | Cloning | 5'TGCTAATGAGTTCACTTGGTATCCC3' |
| PLB-FSII-PF | Cloning | 5'GAATAAATGTGCAGCTTTCAGGT3' |
| PLB-FSII-PR | Cloning | 5'GAAAGGACTGATGGATAAGTGGG3' |
| PLB-LDOX-PF | Cloning | 5'AACGTGTGGCTCTTGATGCCAAATG3' |
| PLB-LDOX-PR | Cloning | 5'GGATTTGATGGATACCACTTGTTGC3' |
| PLB-UFGT-PF | Cloning | 5'CCGTCTGTTTGTATTCTTCTCAT3' |
| PLB-UFGT-PR | Cloning | 5'CTCGATAGTGGACATTATGTGGC3' |
| 0800-PAL-PF | Dual-luc assay | 5'CGGTATCGATAAGCTT TCAGTAGGCCACGGATTGGA3' |
| 0800-PAL-PR | Dual-luc assay | 5'ATCCCCCGGGCTGCAG TTGGTGCAAAAGACAATGAGCA3' |
| 0800-CHI-PF | Dual-luc assay | 5'CGGTATCGATAAGCTT AGTCAGTGTGCCAATGGTGT3' |
| 0800-CHI-PR | Dual-luc assay | 5'ATCCCCCGGGCTGCAGGGTGAGTGAAGAAAAAGTGTT3' |
| 0800-CHS-PF | Dual-luc assay | 5'CGGTATCGATAAGCTTTAGATATGTGCAAAAATAAT3' |
| 0800-CHS-PR | Dual-luc assay | 5'ATCCCCCGGGCTGCAG GGATAATTTTGTGAGGAAT3' |
| 0800-DFR-PF | Dual-luc assay | 5'CGGTATCGATAAGCTTTGGTTTGTGTGACAGGTGCT3' |
| 0800-DFR-PR | Dual-luc assay | 5'ATCCCCCGGGCTGCAGGAATCTCTTAGAAGTGTTGT3' |
| 0800-FLS-PF | Dual-luc assay | 5'CAGCCCGGGGGATCCATGGTGACTGGTATGATGTTGCTTA3' |
| 0800-FLS-PR | Dual-luc assay | 5'TGGCGTCTTCCATGGTGTGTGTGTTTTTGCCTCTTG3' |
| 0800-FSII-PF | Dual-luc assay | 5'CAGCCCGGGGGATCCGAATAAATGTGCAGCTTTCAGGT3' |
| 0800-FSII-PR | Dual-luc assay | 5'TGGCGTCTTCCATGGGTTAAAGAGGTTGGAAAGGTGCAAC3' |
| 0800-LDOX-PF | Dual-luc assay | 5'CAGCCCGGGGGATCCAACGTGTGGCTCTTGATGCCAAATG3' |
| 0800-LDOX-PR | Dual-luc assay | 5'TGGCGTCTTCCATGGTTTGTACGTAAGTGTTGTATTTTGA3' |
| 0800-UFGT-PF | Dual-luc assay | 5'CAGCCCGGGGGATCCCCGTCTGTTTGTATTCTTCTCAT3' |
| 0800-UFGT-PR | Dual-luc assay | 5'TGGCGTCTTCCATGGGAGTTGGTTATTTGTTCAAAAGCTT3' |
| Rt- PAL-PF | Gene expression | 5'TGCTCTATGCCAATCAGTCG3' |
| Rt-PAL-PR | Gene expression | 5'GCGATCAACTACACGAAGCA3' |
| Rt-CHI-PF | Gene expression | 5'GTGTCTGAAATGTGCGTTGG3' |
| Rt-CHI-PR | Gene expression | 5'AGGCGAGGTTGTGAAGAGAA3' |
| Rt-CHS-PF | Gene expression | 5'TGGTCCAGCGATATTGAACA3' |
| Rt-CHS-PR | Gene expression | 5'AGCCCCCATTCTTCTGATTT3' |
| Rt-DFR-PF | Gene expression | 5'ACCCGACCATTGACCATTCT3' |
| Rt-DFR-PR | Gene expression | 5'GTAATCCAAGCCAACCCACG3' |
| Rt-Actin-F | Gene expression | 5' CCAGGCTGTTCAGTCTCTGTAT3' |
| Rt-Actin-R | Gene expression | 5' CGCTCGGTAAGGATCTTCATCA3' |
| PlacZ-CHS -F1 | Y1H | 5'CTCTGATATTGGATCGGAATTCTAGATATGTGCAAAAATAAT3' |
| PlacZ-CHS-R1 | Y1H | 5'CATACAGAGCACATGCCTCGAG GGATAATTTTGTGAGGAAT3' |
| PlacZ-PAL -F1 | Y1H | 5'CTCTGATATTGGATCGGAATTC TCAGTAGGCCACGGATTGGA3' |
| PlacZ-PAL-R1 | Y1H | 5'CATACAGAGCACATGCCTCGAGTTGGTGCAAAAGACAATGAGCA3' |
| PlacZ-CHI -F1 | Y1H | 5'CTCTGATATTGGATCGGAATTC AGTCAGTGTGCCAATGGTGT 3' |
| PlacZ-CHI-R1 | Y1H | 5'CATACAGAGCACATGCCTCGAGGGTGAGTGAAGAAAAAGTGTT3' |
| PlacZ-DFR -F1 | Y1H | 5'CTCTGATATTGGATCGGAATTCTGGTTTGTGTGACAGGTGCT 3' |
| PlacZ-DFR- R1 | 6 Y1H | 5'CATACAGAGCACATGCCTCGAG GAATCTCTTAGAAGTGTTGT3' |
| PlacZ-FLS -F1 | Y1H | 5'CTCTGATATTGGATCGGAATTCATGGTGACTGGTATGATG 3' |
| PlacZ-FLS-R1 | Y1H | 5'CATACAGAGCACATGCCTCGAG TGTGTGTGTTTTTGCCTCTTG3' |
| PlacZ-LDOX-F1 | Y1H | 5'CTCTGATATTGGATCGGAATTCAACGTGTGGCTCTTGATGC3' |
| PlacZ-LDOX-R1 | Y1H | 5'CATACAGAGCACATGCCTCGAGTTTGTACGTAAGTGTTGTA 3' |
| PlacZ-UFGT -F1: | Y1H | 5'CTCTGATATTGGATCGGAATTCCAGATTGGAATTGCCGTCT3' |
| PlacZ-UFGT-R1: | Y1H | 5'CATACAGAGCACATGCCTCGAG GAGTTGGTTATTTGTTCAA3' |
| PlacZ-FSII -F1: | Y1H | 5'CTCTGATATTGGATCGGAATTCGGTATTTAGAACTACATC3' |
| PlacZ-FSII-R1: | Y1H | 5'CATACAGAGCACATGCCTCGAGGTTAAAGAGGTTGGAAAG 3' |
| pB42AD-YAB5F | Y1H | 5'GATTATGCCTCTCCCGAATTCATGTCAAGCATAGATGTAGCA 3' |
| pB42AD-YAB5R | Y1H | 5'GAAGTCCAAAGCTTCTCGAGTCATTTCTTGAATAATGAGGTC 3' |
